# Maternal and Neonatal Complications of Methamphetamine Use during Pregnancy

**DOI:** 10.1155/2021/8814168

**Published:** 2021-04-19

**Authors:** Suthanud Premchit, Nawabhorn Orungrote, Sinart Prommas, Buppa Smanchat, Kornkarn Bhamarapravatana, Komsun Suwannarurk

**Affiliations:** ^1^Department of Obstetrics and Gynecology, Bhumibol Adulyadej Hospital, Royal Thai Air Force, Bangkok, Thailand; ^2^Department of Preclinical Science, Faculty of Medicine, Thammasat University, Ban Klang, Pathumthani, Thailand; ^3^Department of Obstetrics and Gynecology, Faculty of Medicine, Thammasat University, Ban Klang, Pathumthani, Thailand

## Abstract

**Background:**

Methamphetamine abuse has been a significant problem in Thailand. The methamphetamine abuse problem also affects pregnant women. The study of pregnancy outcomes among methamphetamine users during pregnancy is currently limited.

**Objective:**

To determine maternal and neonatal complications among methamphetamine-abusing parturients. *Materials and method*. This historical cohort study was conducted at Bhumibol Adulyadej Hospital (BAH), Bangkok, Thailand, between January 2017 and December 2019. The total number of women was 206 who were equally divided into a study and control group. Pregnant women who tested positive for methamphetamine in urine tests during the intrapartum period were compared to the control group with no history of drug abuse.

**Results:**

Maternal outcomes: gestational hypertension was found to be significantly increased in the study group compared to the control group at 14.6 vs. 1.0% (OR 17.4, 95%CI 2.5-134.3). Preeclampsia with and without severe features were found at higher rates in the study group without statistical significance. There were no eclamptic cases in this study. Neonatal outcomes: preterm birth rate of pregnant women who have tested positive in their urine methamphetamine test was significantly higher than in the control group (33.3%, 11.7%, OR 3.7, 95%CI 1.8-7.7). Average birth weight in the study and control group was 2779.1 ± 486.7 and 3049.5 ± 510 gm, respectively (*p* value < 0.001). Low APGAR score rates of both groups also had no significant difference.

**Conclusion:**

Methamphetamine use during pregnancy increased both maternal and neonatal complications in terms of gestational hypertension, preterm birth, and average birth weight.

## 1. Introduction

The use of amphetamine-type stimulants (ATSs) has been increasing among women worldwide [1]. Cases of pregnant mothers with ATS and maternal/fetal complications are also on the rise [2]. The risk for preterm birth, low birth weight, and small stature of gestational-age infants increases in pregnant women who consume methamphetamine [3].

**Figure 1:**
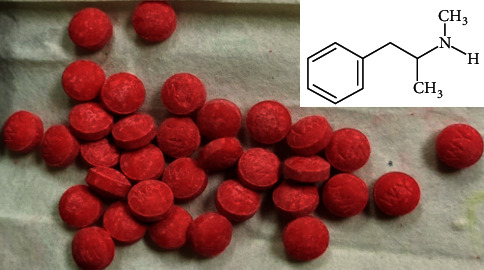
Sample of methamphetamine in Thailand.

ATS is a family of potent central nervous system stimulants composed of amphetamine sulfate and methamphetamine. Amphetamine was first synthesized in 1887. Cases of amphetamine usage in pregnant mothers in Thailand were reported by Thaithumyanon [4]. Methamphetamine (MA) is an amphetamine derivative, composed of a mixture of levo and dextro enantiomers in different proportions by different manufacturers, as shown in Figure 1. MA was introduced in Thailand in the mid-1990s. The illegal use of MA is currently widespread among young adults in Thailand [5]. Both amphetamine and MA are classified as narcotics and, thus, labeled as prohibited substances by Thai law.

In our antenatal care and delivery room at Bhumibol Adulyadej Hospital (BAH), there was an increase in maternal-fetal complications in recent years with pregnant mothers who showed symptoms of methamphetamine withdrawal. Other previous studies in Thailand came from the descriptive retrospective data only, while the current form of methamphetamine was different from the old formulation in the previous data.

The aim of this study was to determine obstetric and neonatal outcomes among methamphetamine-abusing parturient who currently used MA.

## 2. Materials and Method

This retrospective cohort study was conducted at BAH, Bangkok, Thailand, between January 2017 and December 2019. This research protocol was approved by the BAH ethics committee (No. 40/63).

Data were collected from medical records and birth reports from the medical database of the Department of Obstetrics and Gynecology, BAH, during the period of study. Pregnant women who attended the BAH labor room during the period of study with a history of illicit drug (MA, cannabis, cocaine, and opioid) usage during pregnancy or displaying signs and symptoms of MA withdrawal were asked for a urine amphetamine test. MA withdrawal signs and symptoms included agitation, dysphoria, anhedonia, fatigue, and drug craving [3].

Spot urine samples were obtained for MA detection after consent was granted. Urine MA detection was performed using commercial rapid urine tests (Bioline®, Pacific Biotech, BKK, Thailand). Bioline methamphetamine cards are a one-step immunochromatographic assay in which a chemically labeled drug (methamphetamine-protein conjugate) built into the test device competes with methamphetamine. It is used for the qualitative detection of methamphetamine in human urine at a cutoff of 1,000 ng/ml. Singleton pregnant women with positive MA urine tests during the period of study were recruited.

Exclusion criteria were participants with twin pregnancies, fetal anomalies, and underlying health conditions such as pregestational diabetic mellitus and thyroid disease.

Patients with urine MA positive were classified as a study group. The control group was healthy participants who had matching characteristics, namely, age, ethnic group, and mode of delivery during the study period. Data included age, parity, body mass index (BMI), underlying diseases, number of antenatal care visits, and history of drug abuse. The outcomes of pregnancy such as preterm birth, birth weight, Apgar score, and intrapartum complications were also recorded.

Statistical analysis was computerized using Statistical Package for the Social Science software (SPSS Inc., Chicago, IL, USA). Continuous variables were analyzed by Student's *t*-test and mean ± standard deviation (SD). Statistical significance was determined by the probability value of less than 0.05 and 95% confidence interval (CI).

## 3. Results

**Figure 2:**
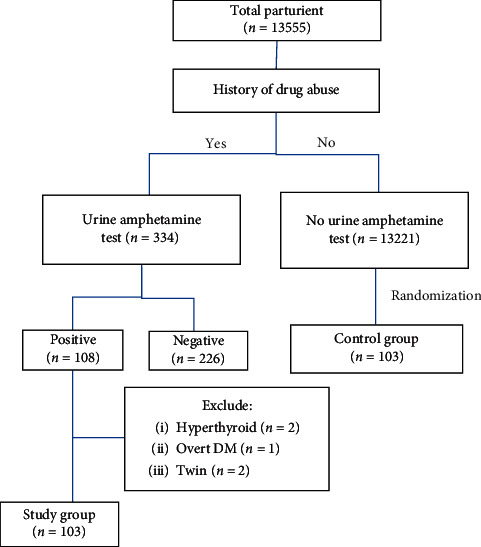
Flow chart of the study and control group.

During the period of study, 206 cases were recruited. Participants were divided into the study and control groups equally as shown in Figure 2. The characteristics of the study and control group are shown in Table 1. Ninety-five percent of cases were Thai. The mean age of study and control groups was 29.2 ± 6.1 and 27.7 ± 6.4 years with no statistical difference. The study group had a lower average number of antenatal care (ANC) visits than the control group with a statistical difference. Half of the study group (48/103) had never undergone any ANC visit. All study groups abused MA via the oral route. The study group had significantly more parity, smoking, alcohol drinking, and sexually transmitted diseases than the control group.

Maternal outcomes of parturient in both groups are summarized and presented in Table 2. Preterm birth rate of the study group was significantly higher than in the control group (33.3% and 11.7%, respectively).

Among complications for pregnancy-induced hypertension, only gestational hypertension was found to be significantly increased in the study group compared to the control group at 14.6 vs. 1.0%, respectively (*p* value < 0.05 with odds ratio 17.4). Preeclampsia with and without severe features were comparable in both the study and the control group, as shown in Table 2. There were no eclampsia cases in this study.

Neonatal outcomes are shown in Table 2. The average birth weight in the study and the control group was 2779.1 ± 486.7 and 3049.5 ± 510 gm, respectively (*p* value < 0.001). Low-APGAR-score cases in both groups showed no significant difference.

## 4. Discussion

Exposure to ATS during pregnancy was reported to cause both obstetric and neonatal complications [1].

Previous studies among methamphetamine abused during pregnancy are summarized and represented in Table 3. The average age of MA pregnant mothers and controls was 28.5 years. Higher number of children, smoking, and alcohol consumption were characteristic features associated with the study group of MA usage during pregnancy than the control group. Almost half of MA group (46.6%) did not attend antenatal care clinics (ANC) compared to 5% of the control group. This finding was in lieu with Thaihumyanon and Thamkhantho's Thailand amphetamine report in 2005 and 2018 which revealed that 80% of pregnant amphetamine users fail to attend ANC [1, 4]. A 2014 US report stated that one quarter of MA pregnant users attend no ANC clinic [6]. MA subjects with no ANC visit could possibly suffer from undetectable ante-, intra-, and postpartum complications.

MA subjects had higher incidences of smoking and alcohol consumption than those in the control group. Alcohol and tobacco were associated with pregnancy outcomes, especially low birth weight [7].

The 2018 annual report from the tobacco control research and knowledge management center (TRC) in Thailand stated that the prevalence of smoking in Thai women was 1.7 percent [8]. In the present study, women with MA abuse concurrently smoked at a rate of 38.8 percent. This tobacco consumption rate in this study was higher than in the general female Thai population.

Another report from the National Statistical Office (NSO) in 2017 reported that 10.6 percent of Thai females consumed alcohol products. In the current study, the percentage of women with MA abuse was similar to that number (10.6 vs. 12.6%) [9]. In the control group, only one percent of the participants consumed alcohol during pregnancy. Data from the Thai NSO were collected for the general Thai female population who were not in the pregnancy stage. However, when those women became pregnant, most would stop drinking except those who consumed MA.

The current study revealed smoking and alcohol consumption in pregnant women with MA at 38.8 and 12.6 percent compared to one and zero percent in the control group, respectively. Data from Thamkhantho's work reported smoking and alcohol drinking at 27.3 and 16.9 percent, respectively [1]. MA addicts had a propensity for using unhealthy substances, namely, tobacco and alcohol [2]. Wright's US study showed 91.5 percent of pregnant women with MA were cigarette smokers but with zero percent alcohol consumption. US Data from the Della Grotta group proposed that MA-addicted pregnant women with decreased MA dosage during pregnancy had higher tendency to use alcohol or tobacco than those who maintained or increased their MA dose [10]. In the present study, the questionnaire did not involve the history of MA dose change. The hypothesis was that the withdrawal symptoms of MA led the MA-consumed pregnant women to seek other substances to suppress their symptoms. Elimination of MA use during pregnancy was a good practice, but counseling and education were needed to halt the seeking of other addictive substances, namely, tobacco and alcohol.

Preterm delivery had multifactorial causes either from maternal or fetal underlying conditions. Vasoconstrictive property of MA during intrautero exposure caused increased risk of preterm birth, low birth weight, and small stature of a gestational-age infant [3]. From the present study, it can be seen that MA-addicted pregnant women had a significantly higher preterm birth rate than the control group, at 33.3 vs. 11.7%, respectively, at *p* < 0.001. MA pregnant women showed a 3.7-fold increased preterm rate compared to the study group. This finding was in line with three other studies from Thailand [1, 4, 11]. These works revealed preterm birth rates in amphetamine-dependent pregnant women ranged between 30.9 and 59.7 percent. The study from the US reported that the rate of preterm birth was between 13 and 50 percent. [2, 6, 12].

Preterm labor could be prevented by cessation of MA and other addictive-substance usage. We recommend the use of a promotion campaign to reach out to MA pregnant women encouraging them to attend ANC clinic. MA-addicted pregnant women should be informed about the complications from MA consumption during the ANC visit and encouraged to stop using it with help from experienced healthcare providers.

MA is one of the sympathomimetic amines. It is not categorized as a major teratogen. MA consumption increases dopamine release and decreases dopamine reuptake. Heart rate and blood pressure increase as a consequence of sympathomimetic effects of MA [7]. MA could traverse the placental barrier and reach the fetus in utero.

Hypertensive disorder during pregnancy (PIH) is a catastrophic event in modern obstetrics. MA enhanced PIH incidence [6]. The current study showed that MA-addicted pregnant women had higher prevalence of gestational hypertension than the control group (14.6 vs. 1%, *p* ≤ 0.001). However, rates of preeclampsia with or without severe features among both groups were comparable.

In the current study, there were no eclampsia cases among MA participants. However, data from Talkathon's report showed that 17 percent of amphetamine-addicted pregnant women came to the labor room with convulsions (eclampsia). The high percentage of eclampsia (17%) in Thamkhantho's study might be the result of a high percentage of amphetamine participants' failure to attend ANC service (79%) [1]. In data from the US, only 0.3 percent of eclampsia was reported in MA-addicted pregnant women [6]. Eclampsia can be prevented by early detection and treatment of preeclampsia with severe features. When many MA-addicted cases did not attend ANC service, many instances of eclampsia could not be prevented.

Vaginal delivery in MA users from our study when compared to the studies of Homsup's group in Thailand and Good's in the US was of similar percentage, ranging from 70 to 88% [6, 12]. The average neonatal birth weight in Homsup's and the present study was 2,797 and 2,779 grams, respectively. In our study, MA-using women had multiparity, low number of ANC visits, and low-birth-weight babies. Half of them had not attended ANC service. They came to the labor room with early symptoms of spontaneous labor onset. Successful vaginal delivery was accomplished.

MA can be collected in the urine in case of repeat usage [13]. The ability to detect MA in urine depended on many factors such as the amount of substance and time of last consumption. This information was not complete due to the limitation of retrospective study.

Limitations of this study included its methodology as a retrospective self-report questionnaire. Urine tests for MA were performed in all pregnant women with history or suspicion of MA consumption. BAH had no policy to perform this urine test in all pregnant women as a routine screening. As a result, the current number of parturient with MA consumption might have been underreported. Other concomitant substance abuse such as opioids which may affect pregnancy outcomes were not routinely investigated because of high cost of consumption for Thai people.

The author recommended a prospective study in illicit-drug-infested areas to collect all necessary data, along with universal MA screening tests in pregnant mothers. It will allow a true understanding of the size of the MA problem in the pregnant population. An MA urine screening test is also recommended in preterm labor or clinical of pregnancy-induced hypertension cases in such an area.

In conclusion, the present retrospective cohort study confirmed that the use of MA during pregnancy significantly increased both maternal and fetal complication, namely, preterm birth delivery and gestational hypertension. The knowledge can be used to help healthcare staff create a plan for MA parturient in anticipation of a high-risk delivery and postdelivery maternal-fetal treatment. Moreover, the results from this study can be used to inform pregnant women during antenatal care to promote substance-free pregnancy.

## Figures and Tables

**Table 1:** Comparison of characteristic features between the study group and control group.

Characteristic	Study (*n* = 103)	Control (*n* = 103)	OR	95% CI	*p* value
Age (years)	29.2 ± 6.1	27.7 ± 6.4	—	—	0.104
<20	4 (3.9)	9 (8.7)	0.42	0.12–1.41	0.251
20–34	81 (78.6)	78 (75.7)	1.18	0.61–2.26	0.739
≥35	18 (17.5)	16 (15.5)	1.15	0.55–2.40	0.851
Thai ethnicity	101 (98)	95 (92.2)	4.25	0.08–20.53	0.52
Multiparity	85 (82.5)	51 (49.5)	4.81	2.54–9.11	<0.001
PPW (kg)	53.8 ± 11.3	56.1 ± 13.5	—	—	0.191
BMI (kg/m^2^)	21.2 ± 3.9	22.3 ± 4.9	—	—	0.097
ANC visit	2.1 ± 2.8	7.7 ± 3.6	—	—	<0.001
No ANC	48 (46.6)	5 (4.7)	17.10	6.42–45.50	<0.001
Alcohol	13 (12.6)	0 (0)	2.14	1.84–2.49	<0.001
Smoker	40 (38.8)	1 (1.0)	2.55	2.09–3.12	<0.001
STD
Syphilis	1 (0.97)	0 (0)	—	—	1.000
Hepatitis B	0 (0)	0 (0)	—	—	—
HIV	5 (4.9)	0 (0)	—	—	0.059

^∗^n (%), PPW: prepregnancy weight, BMI: body mass index, ANC: antenatal care, No ANC: unattended antenatal care, Alcohol: alcoholic drinker, STD: sexually transmitted disease, HIV: human immunodeficiency virus.

**Table 2:** Comparison of pregnancy outcomes between the study and control group.

	Study (*n* = 103)	Control (*n* = 103)	Crude OR	95%CI	*p* value
GA (weeks)	37.0 ± 2.6	38.1 ± 1.7	—	—	<0.001
Preterm birth rate	34 (33.3)	12 (11.7)	3.73	1.80–7.74	<0.001
Late preterm	26 (25.2)	11 (10.7)	2.82	1.33–6.08	0.253
PIH∗
GHT	15 (14.6)	1 (1.0)	17.43	2.51–134.38	<0.001
PE	5 (4.9)	4 (3.9)	1.26	0.32–4.84	0.749
SPE	7 (6.8)	1 (1.0)	7.43	0.89–61.57	0.065
Vaginal delivery	83 (80.6)	72 (69.9)	1.78	0.93–3.40	0.075
Low APGAR	2 (1.9)	1 (1)	2.01	0.18–22.62	0.560
BW (g)	2779.1 ± 486.6	3049.5 ± 510	—	—	<0.001
<2500	28 (27.2)	11 (10.7)	3.12	1.45–6.68	0.004
2500–4000	73 (70.9)	89 (86.4)	0.38	0.18–0.77	0.011
>4000	2 (1.9)	3 (2)	0.66	0.10–4.03	1

GA: gestational age at delivery, PIH: pregnancy-induced hypertension, GHT: gestational hypertension, PE: preeclampsia with nonsevere feature, SPE: preeclampsia with severe feature, Low APGAR: APGAR score equal to and less than 7 at 5 minutes, BW: average birth weight.

**Table 3:** The comparison studies in patients' characteristic and pregnancy outcomes.

	Homsup [11]	Good [12]	Wright [2]	Gorman [6]	Thamkhantho [1]	Thaithumyanon [4]	Present
Case	197	276	60	8542	77	178	103
Year	2017	2010	2015	2014	2018	2005	2020
Country	Thailand	US (CA)	US (HI)	US (LA)	Thailand	Thailand	Thailand
GA (weeks)	37.0 ± 2.3		38 ± 2.1		36.9 ± 2.1	38.1 ± 2.3	37.0 ± 2.6
Age			28.4 ± 6.7		24.3 ± 5.8	23.4 ± 5.2	29.2 ± 6.1
ANC			7.5 ± 4.4				2.1 ± 2.8
No ANC				2,178 (25.5)	61 (79.2)	141 (79.2)	48 (46.6)
Preterm	64 (32.5)	133 (50)	8 (13)	1,999 (23.4)	46 (59.7)	55 (30.9)	34 (33.3)
PIH		48 (17)					
GHT					4 (5.2)		15 (14.6)
PE	10 (5.1)		4 (6.6)	580 (6.8)		11 (6.2)	5 (4.9)
SPE				213 (2.5)			7 (6.8)
Eclampsia				25 (0.3)	5 (6.49)		0 (0)
Vaginal delivery	174 (88.3)	195 (71)	48 (80.3)		65 (84.4)	146 (82.0)	83 (80.6)
Low APGAR	4 (2)	16 (6)					2 (1.9)
BW (kg)	2.8 ± 0.5		3.1 ± 0.5		2.7 ± 0.4	2.7 ± 0.5	2.8 ± 0.5

GA: gestational age at delivery, ANC: antenatal care, PIH: pregnancy-induced hypertension, GHT: gestational hypertension, PE: preeclampsia with nonsevere feature, SPE: preeclampsia with severe feature, Low APGAR: APGAR score equal to and less than 7 at 5 minutes, BW: average birth weight.

## Data Availability

Data are under supervision by the board of ethical committee.

## References

[1] Thamkhantho M. (2018). Obstetric outcomes of amphetamine misapplication duration pregnancy. *Journal of the Medical Association of Thailand*.

[2] Wright T. E., Schuetter R., Tellei J., Sauvage L. (2015). Methamphetamines and pregnancy outcomes. *Journal of Addiction Medicine*.

[3] Robert R., Michael F. G., Charles J. L., Joshua A. C., Thomas R. M., Robert M. S. (2019). *Substance Abuse in Pregnancy Creasy and Resnik’s Maternal-Fetal Medicine*.

[4] Thaithumyanon P., Limpongsanurak S., Praisuwanna P., Punnahitanon S. (2005). Perinatal effects of amphetamine and heroin use during pregnancy on the mother and infant. *Journal of the Medical Association of Thailand = Chotmaihet Thangphaet*.

[5] Sutcliffe C. G., Aramrattana A., Sherman S. G. (2009). Incidence of HIV and sexually transmitted infections and risk factors for acquisition among young methamphetamine users in northern Thailand. *Sexually Transmitted Diseases*.

[6] Gorman M. C., Orme K. S., Nguyen N. T., Kent E. J., Caughey A. B. (2014). Outcomes in pregnancies complicated by methamphetamine use. *American Journal of Obstetrics and Gynecology*.

[7] Cunningham G., Leveno K., Bloom S. (2018). *Teratology, Teratogens, and Fetotoxic Agents Williams Obstetrics*.

[8] (2020).

[9] (2020).

[10] Della Grotta S., LaGasse L. L., Arria A. M. (2010). Patterns of methamphetamine use during pregnancy: results from the infant development, environment, and lifestyle (IDEAL) study. *Maternal and Child Health Journal*.

[11] Homsup P., Phaloprakarn C., Tangjitgamol S., Manusirivithaya S. (2018). Maternal characteristics and pregnancy outcomes among illicit drug-using women in an urban setting. *Taiwanese Journal of Obstetrics and Gynecology*.

[12] Good M. M., Solt I., Acuna J. G., Rotmensch S., Kim M. J. (2010). Methamphetamine use during pregnancy. *Obstetrics & Gynecology*.

[13] Courtney K. E., Ray L. A. (2014). Methamphetamine: an update on epidemiology, pharmacology, clinical phenomenology, and treatment literature. *Drug and Alcohol Dependence*.

